# Glucocorticoid Resistance Syndrome in 2 Patients With Diverse Genotype

**DOI:** 10.1210/jcemcr/luae243

**Published:** 2025-01-23

**Authors:** Tess Battiola, David Viskochil, Kaci Wolken, Marie Couldwell

**Affiliations:** University of Utah Health, Division of Endocrinology, Salt Lake City, UT 84108, USA; University of Utah Health, Division of Medical Genetics, Salt Lake City, UT 84113 USA; Haywood Regional Medical Center, Division of Internal Medicine, Clyde, NC 28721, USA; University of Utah Health, Division of Endocrinology, Salt Lake City, UT 84108, USA

**Keywords:** hypercortisolism, glucocorticoid receptor gene, glucocorticoid resistance syndrome

## Abstract

Glucocorticoid resistance syndrome (GRS) is caused by inactivating pathogenic variants in the glucocorticoid receptor gene *NR3C1*. Reduced glucocorticoid receptor signaling leads to decreased tissue sensitivity to cortisol and resultant biochemical hypercortisolism without the classic clinical features of Cushing syndrome. Patients variably present with signs and symptoms of mineralocorticoid and androgen excess from ACTH overstimulation of the adrenal cortex. Neuropsychiatric symptoms, such as anxiety, depression, anorexia, and insomnia, have also been reported and may be related to CRH excess. Due to the broad clinical spectrum and genetic heterogeneity of the disorder, it remains a diagnostic and treatment challenge. In this report, we describe 2 cases of GRS that highlight the genetic diversity of the condition. Both patients had prominent neuropsychiatric symptoms. While 1 patient had no identifiable variant in the glucocorticoid receptor gene, the other was found to have a novel *NR3C1* variant. Low-dose dexamethasone treatment led to clinical improvement in the patient with negative genetic testing, and the second patient continues to be monitored.

## Introduction

Glucocorticoid resistance syndrome (GRS) is a rare genetic disorder characterized by diminished sensitivity of target tissues to cortisol due to impaired glucocorticoid receptor (hGR) function [1]. It is caused by inactivating pathogenic variants in the hGR gene, *NR3C1*, located on chromosome 5 (5q31.3) [2]. To date, there have been 33 pathogenic variants in *NR3C1* identified in the Human Gene Mutation Database 2021.3. Cases of GRS without a recognizable hGR pathogenic variant have also been reported [3‐5].

Defects in glucocorticoid receptor function result in decreased negative feedback inhibition of the hypothalamic-pituitary-adrenal (HPA) axis leading to increases in CRH, ACTH, and cortisol levels. Excess secretion of CRH and ACTH can induce adrenal hyperplasia and overproduction of mineralocorticoids and/or androgens. The clinical spectrum of GRS is broad, owing to distinct *NR3C1* pathogenic variants and differential activity at the glucocorticoid, mineralocorticoid, and androgen receptors in peripheral tissues [6].

GRS should be suspected when biochemical hypercortisolism is present without classic clinical manifestations of Cushing syndrome. While some patients are asymptomatic, others present with chronic fatigue, anxiety, depression, and weight loss due to excess CRH [7‐10]. Symptoms may also include those related to mineralocorticoid excess such as hypertension and hypokalemic alkalosis or androgen excess such as acne, hirsutism, infertility, precocious puberty, and menstrual irregularities [2]. Other conditions to consider in the differential diagnosis may include mild Cushing syndrome, pseudo-Cushing syndrome, polycystic ovary syndrome, congenital adrenal hyperplasia, and conditions with elevated SHBG.

The diagnosis of GRS involves documentation of biochemical hypercortisolism in the absence of signs or symptoms of Cushing syndrome. Patients often have marked elevations in 24-hour urine-free cortisol (UFC) and failure of cortisol suppression following oral dexamethasone [6]. Evaluation should also include serum or plasma ACTH, aldosterone, renin activity, androgens, and electrolytes. The cornerstone of diagnosis is sequencing of the *NR3C1* gene, which may confirm a loss-of-function pathogenic variant.

## Case Presentation

### Patient A

A 66-year-old slim female was referred to endocrinology in 2021 for hypercortisolism following evaluation by her primary care physician for recent-onset weight loss, worsening anxiety, insomnia, poor memory, and new-onset hypertension and hyperglycemia. She denied any fatigue, muscle weakness, skin changes, abnormal hair growth, acne, or fractures. She had no history of infertility. Hypertension was controlled on lisinopril 20 mg daily, and type 2 diabetes was managed on metformin and sitagliptin. She had no underlying substance use or history of depression.

On physical exam, blood pressure was 133/70 mmHg with a body mass index of 21 kg/m^2^. She was anxious. She had no moon facies, dorsocervical fat pads, central obesity, abdominal striae, skin atrophy, or hirsutism. Muscle mass and strength were normal.

### Patient B

A 43-year-old female was referred to endocrinology in 2019 for evaluation of an adrenal incidentaloma. She reported a 50-pound weight loss, fatigue, and worsening anxiety and depression. Menses were absent following a hysterectomy.

Her medical history was notable for depression and previous miscarriages. She required fertility treatment for 1 pregnancy. She had no history of diabetes or hypertension. Her daughter, who was found to have elevated 24-hour UFC, had a history of irregular menses.

On exam, blood pressure was 110/76 mmHg with a body mass index of 24 kg/m^2^. Her abdomen was slightly protuberant without striae. She had no evidence of moon facies, dorsocervical fat pads, skin atrophy, abdominal striae, excess facial hair, male pattern hair loss, acne, or myopathy.

## Diagnostic Assessment

### Patient A

Laboratory evaluation was notable for morning hypercortisolism with inappropriately normal ACTH, elevated UFC, elevated late-night salivary cortisol levels, and abnormal high-dose dexamethasone suppression test (Table 1). Serum potassium, testosterone, androstenedione, dehydroepiandrosterone-sulfate, aldosterone, and plasma renin activity levels were all within the reference ranges (Table 1).

**Table 1.** Results of biochemical and genetic evaluation for patient A and patient B

	Reference range	Patient A	Patient B
Serum potassium	3.3-5.0 mmol/L	**3.9-4.2 mmol/L**	**3.8-4.3 mmol/L**
Am plasma cortisol	10-20 µg/dL (275.9-551.7 nmol/L)	12/2021: **22.6 µg/dL** (623.4 nmol/L) 6/2022: **21.9 µg/dL** (604.1 nmol/L) 12/2022: **20.8 µg/dL** (573.8 nmol/L)	8/2019: **32.7 µg/dL** (902.0 nmol/L) 3/2020: **33.9 µg/dL** (935.2 nmol/L) 4/2021: **20.6 µg/dL** (568.3 nmol/L)
UFC	<45.0 µg/24h (<124.1 nmol/24 hours)	11/2021: **240.0 µg/24h** (662 nmol/24 hours) 1/2022: **147.9 µg/24h** (408.0 nmol/24 hours)	10/2018: **508.0 µg/24h** (1401.3 nmol/24 hours) 2/2019: **197.3 µg/24h** (544.3 nmol/24 hours) 11/2019: **176.4 µg/24h** (486.6 nmol/24 hours) 1/2020: **153.6 µg/24h** (423.7 nmol/24 hours)
Late-night salivary cortisol	<0.112 µg/dL (<3.1 nmol/L)	1/2022: **0.269 µg/dL** (7.4 nmol/L) 1/2022: **0.478 µg/dL** (13.2 nmol/L) 2/2023: **0.434 µg/dL** (12.0 nmol/L)	1/2020: **0.124 µg/dL** (3.4 nmol/L) 1/2020: **0.113 µg/dL** (3.1 nmol/L) 4/2020: **0.108 µg/dL** (3.0 nmol/L)
Am cortisol	<1.8 µg/dL (<49.7 nmol/L)	4/2022: **2.2 µg/dL** (60.7 nmol/L) post 8-mg dexamethasone	1/2020: **12.1 µg/dL** (333.8 nmol/L) post 1-mg dexamethasone
Dexamethasone level		640.0 µg/dL (17,657.6 nmol/L) (expected range 1600-2850 µg/dL or 44,144-78,631.5 nmol/L)	247.7 µg/dL (6834.0 nmol/L) (expected range 140-295 µg/dL or 3862.6-8139.1 nmol/L)
Am ACTH	7.2-63.3 pg/mL (26.4-232.4 pmol/L)	1/2022: **17.5 pg/mL** (64.2 pmol/L) 6/2022: **17.6 pg/mL** (64.6 pmol/L) 2/2022: **30.5 pg/mL** (112.0 pmol/L)	10/2018: **22 pg/mL** (80.8 pmol/L) 8/2019: **25 pg/mL** (91.8 pmol/L) 3/2020: **24.9 pg/mL** (91.4 pmol/L) 6/2022: **21.1 pg/mL** (77.5 pmol/L)
Aldosterone	4.0-31.0 µg/dL (110.3-855.2 nmol/L)	12/2022: **6.4 µg/dL** (176.6 nmol/L)	4/2021: **22.9 µg/dL** (631.7 nmol/L)
Renin activity	0.2-4.0 ng/mL/h (0.55-11.0 nmol/L/h)	12/2022: **0.4 ng/mL/h** (1.10 nmol/L/h)	
Total testosterone	5-32 ng/dL (0.17-1.1 nmol/L)	6/2022: **13 ng/dL** (0.45 nmol/L)	4/2021: **14 ng/dL** (0.49 nmol/L) 7/2022: **21 ng/dL** (0.73 nmol/L)
Androstenedione	0.13-0.82 ng/mL (0.45-2.8 nmol/L)	6/2022: **0.369 ng/mL** (1.28 nmol/L)	4/2021: **0.528 ng/mL** (1.83 nmol/L)
DHEA-S	9-246 µg/dL (24.8-678.6 μmol/L)	6/2022: **188 µg/dL** (518.6 μmol/L)	4/2021: **53 µg/dL** (146.2 μmol/L)
17-OH progesterone	<206 ng/dL (<7.1 nmol/L)		10/2018: **40.0 ng/dL** (1.4 nmol/L) 4/2021: **336.0 ng/dL** (11.6 nmol/L)
11-deoxycortisol	<32 ng/dL (<1.1 nmol/L)		4/2021: **33.7 ng/dL** (1.2 nmol/L)
Genetic testing		2/2023: **Negative for *NR3C1* genetic variants**	12/2021: **Heterozygous for *NR3C1* c.1322G > C**

Bold letters indicate results in metric units of measurements. Abbreviations: 17-OH progesterone, 17-hydroxyprogesterone; Am, morning; DHEA-S, dehydroepiandrosterone-sulfate; UFC, 24-hour urine free cortisol.

**Figure 1.**
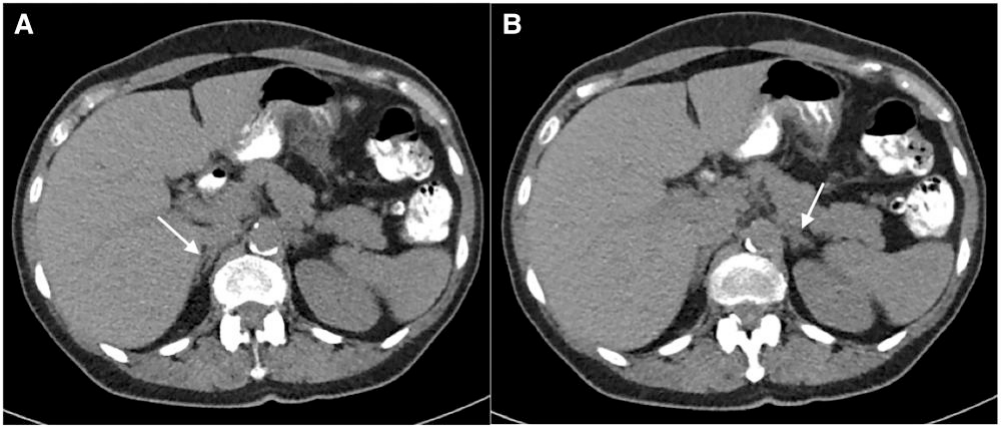
(A) Patient A's CT abdomen and pelvis noncontrast showing normal right adrenal gland. (B) Patient A's CT abdomen and pelvis noncontrast showing normal left adrenal gland. Abbreviation: CT, computed tomography.

Imaging studies, including magnetic resonance imaging of the pituitary and computed tomography scans of the chest, abdomen, and pelvis were normal (Fig. 1A and 1B). Inferior petrosal sinus sampling (IPSS) was nondiagnostic (Table 2). Full-body Cu-64 DOTATATE-positron emission tomography scan showed increased uptake in a left thyroid nodule, of which a fine needle aspiration biopsy was benign. Dual-energy X-ray absorptiometry scan was normal.

**Table 2.** Results of inferior petrosal sinus sampling in patient A

		ACTH levels		
Time		Right inferior petrosal sinus	Left inferior petrosal sinus	Peripheral site
Baseline 1	11:25	39.2 pg/mL (143.9 pmol/L)	<1.5 pg/mL (<5.5 pmol/L)	<1.5 pg/mL (<5.5 pmol/L)
Baseline 2	11:31	28.4 pg/mL (104.3 pmol/L)	<1.5 pg/mL (<5.5 pmol/L)	<1.5 pg/mL (<5.5 pmol/L)
DDAVP injection	11:33			
Post 2-min	11:35	35.4 pg/mL (130.0 pmol/L)	2.0 pg/mL (7.3 pmol/L)	<1.5 pg/mL (<5.5 pmol/L)
Post 5-min	11:38	23.8 pg/mL (87.4 pmol/L)	1.9 pg/mL (7.0 pmol/L)	<1.5 pg/mL (<5.5 pmol/L)
Post 10-min	11:43	29.5 pg/mL (108.3 pmol/L)	<1.5 pg/mL (<5.5 pmol/L)	<1.5 pg/mL (<5.5 pmol/L)
Central/peripheral ACTH	>23 pg/mL (>84.4 pmol/L)			
R IPS ACTH/L IPS ACTH	>1.4 pg/mL (>5.1 pmol/L)			

Abbreviations: DDAVP, desmopressin acetate; L IPS, left inferior petrosal sinus; R IPS, right inferior petrosal sinus.

The patient's persistent ACTH-dependent hypercortisolism without clinical stigmata of Cushing syndrome, lack of an identifiable pituitary or ectopic source of ACTH, and no compelling evidence to substantiate a diagnosis of pseudo-Cushing syndrome made her clinical picture suggestive for GRS. *NR3C1* sequencing did not identify a pathogenic variant.

### Patient B

Laboratory evaluation revealed morning hypercortisolism, elevated UFC on several occasions, nonsuppressible morning plasma cortisol following oral dexamethasone 1 mg, and elevated late-night salivary cortisol levels. Plasma ACTH was inappropriately normal. Serum potassium, total testosterone, dehydroepiandrosterone-sulfate, and androstenedione were normal. On initial testing, the serum 17-hydroxyprogesterone level was normal but was later found to be elevated along with 11-deoxycortisol (Table 1).

**Figure 2.**
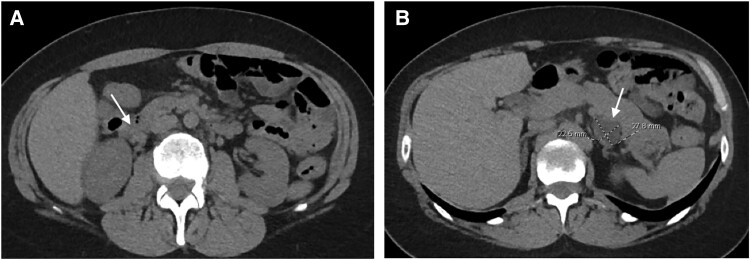
(A) Patient B's CT abdomen/pelvis noncontrast showing hyperplasia of the right adrenal gland. (B) Patient B's CT abdomen/pelvis noncontrast showing a left adrenal nodule. Abbreviation: CT, computed tomography.

Computed tomography imaging showed right adrenal gland hyperplasia and a left adrenal nonenhancing mass measuring approximately 2.1 cm, consistent with a benign adenoma (Hounsfield units −10) (Fig. 2A and 2B). Magnetic resonance imaging of the pituitary did not reveal any lesion. IPSS showed a gradient for ACTH secretion with lateralization to the right (Table 3). The patient underwent transsphenoidal exploration and unilateral pituitary resection. ACTH staining of the pituitary tissue by immunohistochemistry was negative. The left adrenal adenoma remained unchanged on repeat imaging. Sequence and copy number variation analysis of *NR3C1* using proprietary software (Blueprint Genetic Glucocorticoid Deficiency Panel) demonstrated the patient to be heterozygous for a variant in the *NR3C1* gene c.1322G > C, p.(Cys441Ser). This missense variant results in a substitution of cysteine to serine at nucleotide position 1322, a highly conserved amino acid in the zinc finger DNA-binding domain.

**Table 3.** Results of inferior petrosal sinus sampling in patient B

		ACTH levels		
Time		Right inferior petrosal sinus	Left inferior petrosal sinus	Peripheral sit
Baseline 1	11:10	1.4 pg/mL (5.1 pmol/L)	6.8 pg/mL (25.0 pmol/L)	2.6 pg/mL (9.5 pmol/L)
Baseline 2	11:16	7.9 pg/mL (29.0 pmol/L)	1.0 pg/mL (3.7 pmol/L)	2.3 pg/mL (8.4 pmol/L)
CRH injection	11:18			
Post 2-min	11:20	67.8 pg/mL (248.9 pmol/L)	1.9 pg/mL (7.0 pmol/L)	2.6 pg/mL (9.5 pmol/L)
Post 5-min	11:23	421.5 pg/mL (1547.3 pmol/L)	5.8 pg/mL (21.3 pmol/L)	7.1 pg/mL (26.1 pmol/L)
Post 10-min	11:28	193.3 pg/mL (709.6 pmol/L)	13.9 pg/mL (51.0 pmol/L)	14.8 pg/mL (54.3 pmol/L)
Central/peripheral ACTH	>23 pg/mL (84.4 pmol/L)			
R IPS ACTH/L IPS ACTH	>1.4 pg/mL (5.1 pmol/L)			

Abbreviations: L IPS, left inferior petrosal sinus; R IPS, right inferior petrosal sinus.

## Treatment

### Patient A

Patient A was started on low-dose oral dexamethasone, 0.25 mg daily, for management of her symptoms.

### Patient B

After a shared decision-making discussion, glucocorticoid therapy was deferred.

## Outcome and Follow-up

### Patient A

Within 1 month of starting treatment, anxiety and insomnia rapidly improved. She had gained 6 pounds and both hypertension and diabetes were controlled.

### Patient B

Symptoms remain stable. Patient B's daughter has not yet undergone genetic testing.

## Discussion

We report 2 cases of GRS that highlight its clinical and genetic heterogeneity. The first case (patient A) describes a patient with neuropsychiatric symptoms who was found to have persistently elevated cortisol levels in an ACTH-dependent manner without classic Cushingoid features or evidence of mineralocorticoid/androgen excess. No central or peripheral source of ACTH was identified, and she had no clear alternate explanation for persistent hypercortisolism. Thus, her presentation is most consistent with GRS. However, mutational analysis did not reveal a pathogenic variant in the coding region of *NR3C1*, the hGR gene. There have been other reports of GRS in the absence of hGR pathogenic variants [3‐5]. These patients are thought to have pathogenic variants in other steps or elements of the glucocorticoid signaling pathway, such as postreceptor activity, nuclear cofactors, and other molecular determinants of glucocorticoid tissue sensitivity. The pathogenesis for GRS in this case remains unclear, but we can speculate that the functional impairment in glucocorticoid signaling did not impact androgen and mineralocorticoid production, leading to a lack of androgen or mineralocorticoid excess. Additional research is needed to elucidate the molecular pathogenesis of GRS in patients with negative *NR3C1* sequencing.

We present a second case (patient B) of persistent biochemical hypercortisolism in a patient without clinical stigmata of Cushing syndrome. In contrast to patient A, patient B had features of hyperandrogenism including infertility and elevations in 17-hydroxyprogesterone and 11-deoxycortisol, as well as adrenal hyperplasia on computed tomography imaging. Genetic analysis revealed heterozygosity for a monoallelic missense variant that has not been previously reported. In silico models of the G > C missense mutation at nucleotide position 1322 predict the variant to be deleterious to protein function. While functional assessment of the variant is not available, we presume that this variant is pathogenic.

The genetic variation among patients with GRS may explain the broad clinical spectrum of the disorder. Pathogenic monoallelic mutations in the *NR3C1* gene may result from haploinsufficiency or dominant-negative effects. Both mechanisms can be associated with missense variants, such as the one identified in patient B [5]. This mutation occurs in the proximal box of the DNA-binding domain, replacing 1 of the 4 critical cysteine residues—required for zinc binding—with a serine residue. We propose that this disrupts the zinc binding site, impairing the receptor's ability to bind glucocorticoid-response elements and altering transcriptional regulation of downstream genes involved in glucocorticoid signaling. The exact mechanism of pathogenicity, namely haploinsufficiency or dominant-negative activity, is dependent on the transcriptional changes in glucocorticoid-response elements-related genes and their effect on the cortisol-hGR complex. Further functional study of the DNA-binding and transactivation properties of this variant is required to precisely characterize its pathogenicity and clinical manifestations.

We suggest that *NR3C1* sequencing be completed in patients with persistent biochemical hypercortisolism in the absence of clinical signs/symptoms of Cushing syndrome and in patients with a known or suspected family history of glucocorticoid resistance. While GRS is a rare disorder, we urge providers to maintain a high index of suspicion for these cases to avoid unnecessary, invasive testing. If *NR3C1* sequencing had been completed earlier in the clinical course of Patient B, invasive testing with IPSS and transsphenoidal exploration/resection could have been avoided.

Management of GRS is aimed at suppression of the HPA axis to reduce CRH and ACTH-mediated hypercortisolism, mineralocorticoid, and/or androgen excess. Dexamethasone can be titrated to symptoms and biochemical endpoints [1]. In patients with identifiable *NR3C1* pathogenic variants, the utility of oral glucocorticoids in symptom management may be dependent upon the functional characterization of the mutation.

## Learning Points

- GRS should be suspected in patients with biochemical hypercortisolism without the classic clinical features of Cushing syndrome.
- The diagnostic evaluation for GRS involves documentation of biochemical hypercortisolism with elevated or inappropriately normal ACTH levels, reflecting central resistance to glucocorticoid signaling, testing for mineralocorticoid and androgen excess, and genetic sequencing of the *NR3C1* gene.
- Further study is needed to explore the molecular pathogenesis of GRS in patients with normal *NR3C1* gene sequencing.
- Patients may benefit clinically from suppression of the HPA axis using an oral, long-acting corticosteroid, though the utility of treatment may depend on the functional characterization and specific pathogenesis of their glucocorticoid resistance defect.

## Data Availability

Data sharing is not applicable to this article as no datasets were generated or analyzed during the current study.
